# Chitosan-Alginate Biocomposite Containing Fucoidan for Bone Tissue Engineering

**DOI:** 10.3390/md12010300

**Published:** 2014-01-16

**Authors:** Jayachandran Venkatesan, Ira Bhatnagar, Se-Kwon Kim

**Affiliations:** 1 Marine Bioprocess Research Center, Department of Chemistry, Pukyong National University, Busan 608-737, Korea; E-Mail: venkatjchem@pknu.ac.kr; 2 Nanotheranostics Laboratory, Centre for Cellular and Molecular Biology, Hyderabad 500-007, India; E-Mail: ira@ccmb.res.in

**Keywords:** chitosan, alginate, fucoidan, bone tissue engineering, biomaterials

## Abstract

Over the last few years, significant research has been conducted in the construction of artificial bone scaffolds. In the present study, different types of polymer scaffolds, such as chitosan-alginate (Chi-Alg) and chitosan-alginate with fucoidan (Chi-Alg-fucoidan), were developed by a freeze-drying method, and each was characterized as a bone graft substitute. The porosity, water uptake and retention ability of the prepared scaffolds showed similar efficacy. The pore size of the Chi-Alg and Chi-Alg-fucoidan scaffolds were measured from scanning electron microscopy and found to be 62–490 and 56–437 µm, respectively. *In vitro* studies using the MG-63 cell line revealed profound cytocompatibility, increased cell proliferation and enhanced alkaline phosphatase secretion in the Chi-Alg-fucoidan scaffold compared to the Chi-Alg scaffold. Further, protein adsorption and mineralization were about two times greater in the Chi-Alg-fucoidan scaffold than the Chi-Alg scaffold. Hence, we suggest that Chi-Alg-fucoidan will be a promising biomaterial for bone tissue regeneration.

## 1. Introduction

Bone, a complex and hierarchical tissue with a major portion made up of hydroxyapatite (HA) and collagen, plays a major role in the structural framework, mineral deposition, pH regulation and mechanical support. Bone defects or damages are possible in a number of ways, including motor accidents, birth defects and chronic diseases. Over 2.2 million-bone graft procedures are performed annually worldwide [1,2]. Several materials or treatment options are available to reconstruct bone defects, such as autograft, allograft, xenograft and synthetic graft. Autograft and allograft techniques are ideal bone graft procedures; however, a few concerns (such as problems of donor site morbidity, the availability of bone grafts for use with the autograft technique and the associated risk of transmissible diseases, for example, acquired immune deficiency syndrome (AIDS) or hepatitis in the allograft) exist. Recently, considerable attention has been given to synthetic tissue engineering scaffolds for the construction of artificial bone. Such synthetic bone grafts should be biocompatible, biodegradable, osteoconductive, osteoinductive and structurally similar to bone, with excellent mechanical strength, easy to handle and cost effective [2,3].

Chitin is a natural polysaccharide, and it is the most important polymer after cellulose. It occurs in the exoskeleton of arthropods or in the cells walls of fungi and yeast. An important derivative of chitin is chitosan. It is obtained by partial deacetylation of chitin using a chemical method (concentrated NaOH) or by enzymatic hydrolysis [4]. Chitosan (Chi) is composed of repeating units of d-glucosamine and *N*-acetyl glucosamine linked in a β (1-4) manner. Chitosan possesses excellent biocompatibility, biodegradation, antimicrobial activity and low immunogenicity. It can be molded into various forms (gels, membranes, sponges, beads and scaffolds) and has an exceptional pore forming ability for potential applications in tissue engineering, drug delivery and wound healing [4,5]. Chitosan has been combined with a variety of biopolymers and bioceramic systems, such as alginate, hyaluronic acid, amylopectin, carbon nanotubes, poly(methyl methacrylate), polylactic acid, growth factors, HA and calcium phosphate [6,7,8,9,10,11,12,13]. Alginate (Alg) is an anionic linear copolymer that is made up of homopolymeric blocks of both (1-4)-linked β-d-mannuronate and α-l-guluronate residues. These homopolymeric blocks are covalently linked together in sequences that include blocks of alternating α-l-guluronate-β-d-mannuronate copolymer. Alginate is commonly isolated from marine seaweed. Similar to chitosan, alginate is also an exceptional biomaterial for bone tissue engineering, due to its biocompatibility, biodegradability, non-antigenicity, encapsulation capacity, chelating ability and ability to be cast in different forms, such as gels, microspheres, foams, fibers and sponges. Alginate scaffolds are often used for delivering materials, such as bone morphogenetic protein-2 (BMP-2) and mesenchymal stem cells (MSC) to the defective area for repairing the tissues [14,15]. Chi-Alg composites have been widely used for drug delivery and protein delivery [16,17,18,19,20,21,22], wound healing [23,24,25], tendon and ligament tissue engineering [26] and intervertebral tissue engineering [27]. In fact, a few reports are also available on a calcium-based composite using Alg-*N*-succinyl-chitosan for bone tissue regeneration [28]. Apart from this, Chi-Alg-MSC-BMP-2 composite was used to generate new bone [29].

Fucoidan is a sulfated polysaccharide that contains l-fucose and sulfate. It is commonly found in marine brown seaweeds. Fucoidan can increase the level of alkaline phosphatase (ALP), type-1 collagen expression, osteocalcin and BMP-2 and even helps in mineral deposition associated with bone mineralization [30,31]. In addition, fucoidan treatment enhanced the expression of ALP, type-1 collagen, Runt-related transcription factor 2 (Runx-2), osteopontin and osteocalcin in human adipose-derived stem cells. It also promoted osteogenic differentiation in human amniotic fluid stem cells, which suggested that it is a potential candidate for bone tissue regeneration [32]. Composite containing polycaprolactone-fucoidan showed excellent cellular proliferation and mineralization [33,34]. Around 30% enhanced mineral deposition was observed in fucoidan containing composite. This is mainly because of the presence of fucoidan in the composite scaffold. [31]. Moreover, Chi-fucoidan composite film showed significant wound dressing ability *in vitro* and *in vivo* [35].

Hence, considering the biocompatibility, biodegradation, antibacterial nature, film-forming ability and induction of osteogenic differentiation by fucoidan, Chi and Alg, we aimed to report the synthesis and characterization of the newly developed Chi-Alg biocomposite containing fucoidan for bone tissue engineering.

## 2. Results and Discussion

### 2.1. General Observation

**Figure 1:**
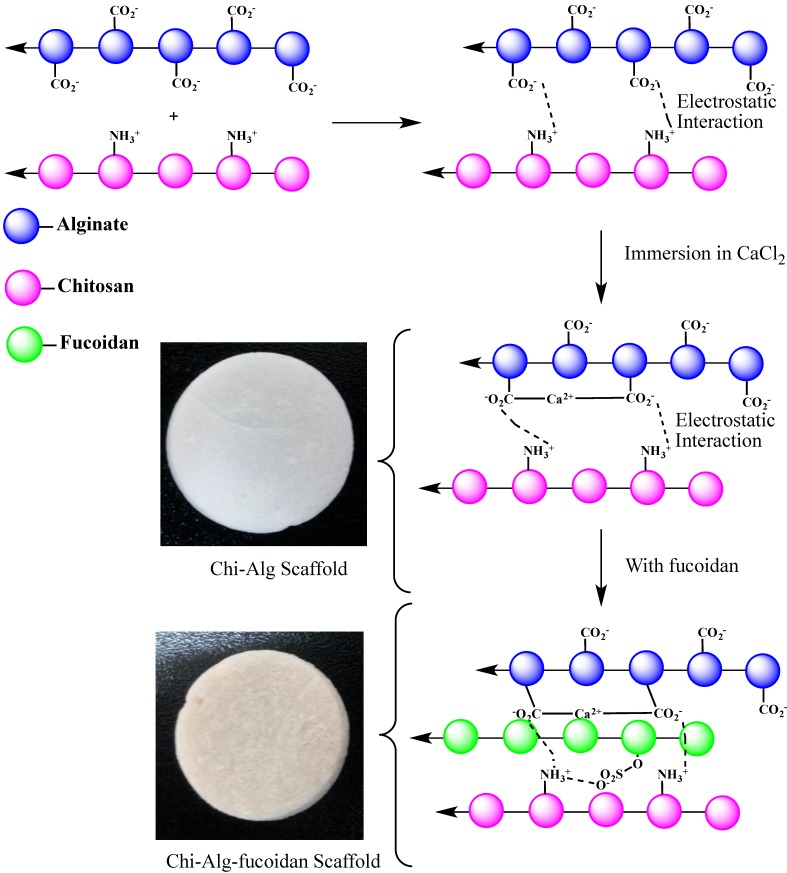
Graphical representation of the chemical interaction of fucoidan incorporated alginate (Alg)-chitosan (Chi) composite scaffolds.

In this study, scaffolds were fabricated by a freeze-drying method. The Chi-Alg and Chi-Alg-fucoidan scaffolds were found to be stiff and inelastic. Chi-Alg scaffolds were observed in a colorless state, whereas Chi-Alg-fucoidan scaffolds obtained a pale brown color, which is due to the dispersion of fucoidan in the composites. By the visual observation (Figure 1), fucoidan was uniformly dispersed in the Chi-Alg composite, and no agglomeration was observed. The preparation procedure is graphically sketched and shown in Figure 1.

### 2.2. Fourier Transform-Infrared Spectroscopy

**Figure 2:**
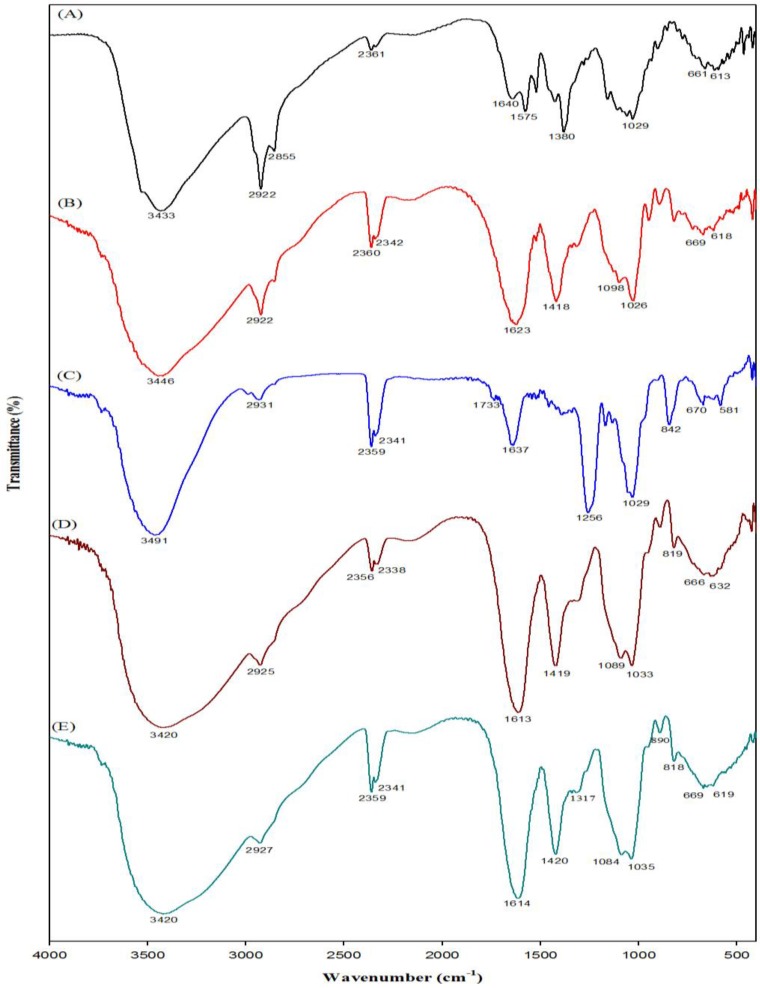
Fourier transform infrared spectrum of (**A**) chitosan, (**B**) alginate, (**C**) fucoidan, (**D**) Chi-Alg and (**E**) Chi-Alg-fucoidan.

Alginate is an anionic polymer that possesses the ability to form strong electrostatic interaction with cationic polymers. In this case, the cationic charged amine group of the chitosan unit interacted electrostatically with the negatively charged alginate to form a polyelectrolyte mixture. The addition of fucoidan in the Chi-Alg may make more complex ionic interactions possible. Several studies suggested that hydrogen bonding or ion-ion pair interaction between the components usually increases the uniform dispersion [36]. These molecular chemical interactions between Chi-Alg and Chi-Alg-fucoidan were studied by FT-IR. FT-IR spectra were used to confirm the functional groups and interactions of chitosan, alginate, fucoidan, Chi-Alg and Chi-Alg-fucoidan scaffolds, and the spectra are depicted in Figure 2.

The alginate spectrum shows the characteristic peak at 1623 cm^−1^, which corresponds to the carboxylate group (C=O). In addition, a strong intense peak was observed at 3446 cm^−1^, corresponding to the –OH group. The symmetric stretching frequency of the carboxyl group was observed at 1418 cm^−1^, whereas 1098–1026 cm^−1^ shows the asymmetric stretching frequency. Chitosan shows vibration at 3433 cm^−1^ (–OH and N-H stretching vibrations), 2855 cm^−1^ (C-H stretch), 1640 cm^−1^ (amide I), 1575 cm^−1^ (N-H bending of amine) and 1029 cm^−1^ (skeletal vibration of C-O stretching frequency).

On the other hand, in the case of Chi-Alg, an intense peak was observed at 1613 cm^−1^, corresponding to the superposition of the bands assigned to the carboxylate group of alginate and the amine group of chitosan. The interaction from electrostatic interaction between the carboxylate group of alginate and the amine group of chitosan forms a polyelectrolyte complex. The results are consistent with previous studies [37]. The lower stretching frequency in –OH was observed from 3433 cm^−1^ to 3420 cm^−1^. This suggests that intermolecular hydrogen bonds exist in the chitosan-alginate system [36].

Fucoidan exhibits its characteristic bands at 1210–1280 cm^−1^ assigned to the (S=O) group and a sharp band at 842 cm^−1^ assigned to the sulfate group in fucoidan [38]. The peaks at 3491 cm^−1^ corresponded to the –OH group on the fucoidan moiety. The corresponding stretching frequency of fucoidan is not clearly visible in the Chi-Alg-fucoidan composite. This may be due to the fact that the level of fucoidan in the Chi-Alg-fucoidan scaffold was too small to be detected.

### 2.3. Porosity of the Scaffolds

The porosity of the prepared scaffolds was measured through the liquid displacement method using ethanol. The results suggest that the porosity of the scaffolds are >90%. The porosity of the Chi-Alg and Chi-Alg-fucoidan scaffolds were measured as 94.5% ± 0.5% and 94.9% ± 0.2%, respectively. Greater than 90% total porosity was observed for the polymeric scaffold, which could be an added advantage for tissue engineering purposes [39]. This high degree of porosity would allow cells to migrate into and populate within the scaffold.

### 2.4. Water Uptake and Retention Ability of the Scaffolds

**Figure 3:**
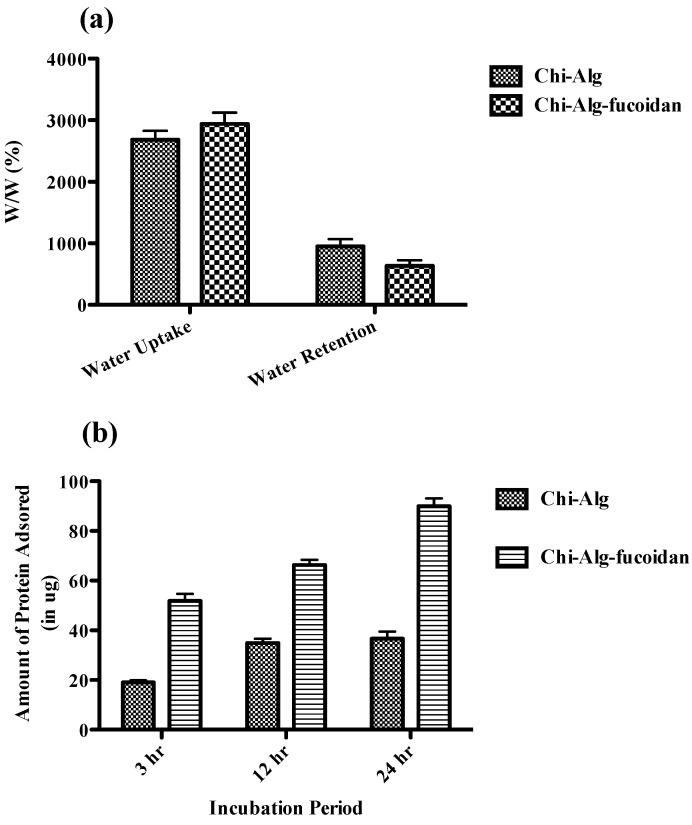
(**a**) Water uptake and retention of Chi-Alg and Chi-Alg-fucoidan composite scaffolds after 24 h. (**b**) Protein adsorption studies of the Chi-Alg and Chi-Alg-fucoidan scaffolds in DMEM containing FBS at 37 °C at different intervals of 3 h, 12 h and 24 h. The values are the mean ± SD of a minimum of three replicates.

The water uptake ability of the scaffolds is measured by the swelling behavior of the scaffold in phosphate buffer saline (PBS) solution. The water uptake and retention ability of the Chi-Alg and Chi-Alg-fucoidan were studied by immersing these scaffolds in 1× PBS solution (Figure 3a). The results showed that there are differences in the swelling behavior among the scaffolds, where the water uptake ability of the Chi-Alg-fucoidan scaffold was higher when compared to Chi-Alg. It has been reported earlier that alginate absorbs water quickly and holds 200–300 times its own weight of water [3]. The addition of negatively charged fucoidan increases the availability of free functional groups in the Chi-Alg-fucoidan system. Hence, the swelling behavior of Chi-Alg-fucoidan is higher compared to the Chi-Alg scaffold. The surface generally increases upon swelling of the scaffold, which is suitable for more cell adhesion and infiltration. Hence, as the Chi-Alg-fucoidan scaffolds exhibited increased swelling, the presence of fucoidan in the scaffolds would exhibit more surface area. The water retention ability of the Chi-Alg-fucoidan scaffold was comparatively less than the Chi-Alg scaffold. This may be due to the fact that unbound water molecules are easily removed from the surface of Chi-Alg-fucoidan scaffold.

### 2.5. Protein Adsorption Efficiency

The study of the protein adsorption of the prepared scaffold plays a significant role in *in vivo* study. Proteins, including fibronectin, vitronectin and other signaling molecules, can be adsorbed by the scaffolds from the circulating body fluids, which facilitate cell adhesion, proliferation and differentiation. The amount of adsorbed protein on the Chi-Alg and Chi-Alg-fucoidan scaffolds was measured with respect to time. The scaffolds were incubated with Dulbecco’s Modified Eagle’s Medium (DMEM) containing 10% Fetal Bovine Serum (FBS), and as shown in Figure 3b, Chi-Alg-fucoidan showed increased protein adsorption from the initial period of incubation. Increasing the incubation period further enhanced the protein adsorption, and the Chi-Alg-fucoidan scaffold showed three times more protein adsorption when compared to the Chi-Alg scaffold (Figure 3b). The negatively charged sulfate group in fucoidan might be electrostatically attracted to the positively charged amino acid in the FBS solution [40].

### 2.6. *In vitro* Biodegradation Behavior

*In vitro* biodegradation is a crucial parameter to be considered in bone tissue engineering. The biodegradation of scaffolds provides space for tissue growth and matrix deposition. In the present study, no difference was observed in the biodegradation in Chi-Alg (15%) and Chi-Alg-fucoidan (15%) at 24 h. However, higher degradation was observed in the Chi-Alg-fucoidan scaffold (40%) compared to Chi-Alg (15.7%) at 72 h. This might be because of the electrostatic interactions between chitosan and fucoidan, as well as the weak ionic bonding forces between fucoidan and calcium in PBS.

### 2.7. Scanning Electron Microscopy and Optical Microscopy Analysis

**Figure 4:**
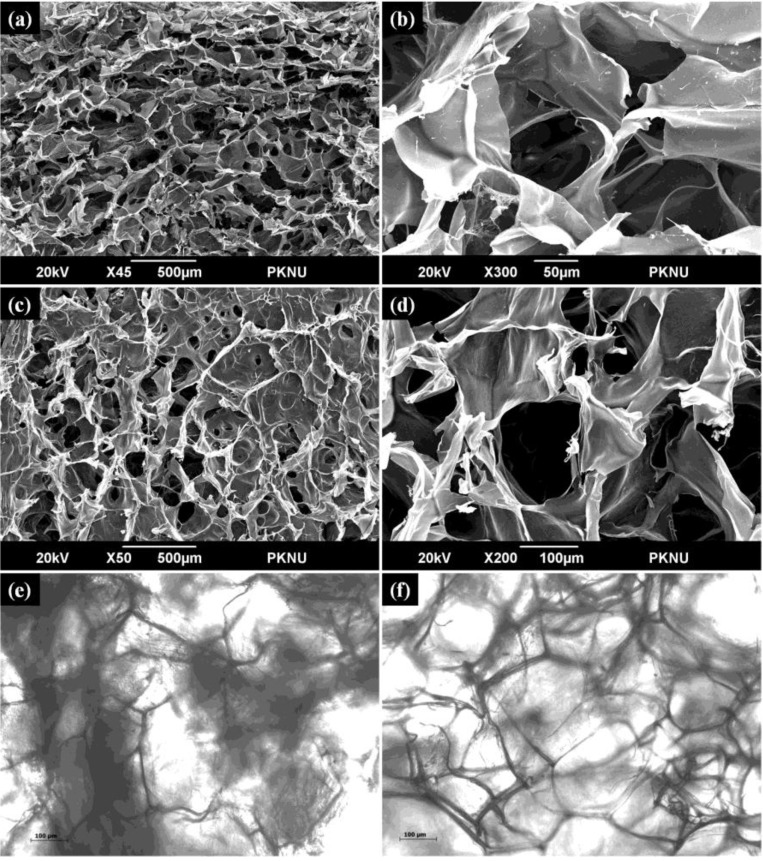
High and low magnification SEM micrographs of (**a**, **b**) Chi-Alg and (**c**, **d**) Chi-Alg-fucoidan; and optical microscopy images of (**e**) Chi-Alg and (**f**) Chi-Alg-fucoidan.

The surface morphology, pore distribution and pore size of Chi-Alg and Chi-Alg-fucoidan were examined using scanning electron microscopy analysis (Figure 4). The pore sizes of the scaffolds were directly measured in a scanning electron micrograph, and all the prepared scaffolds were found to be highly porous with a pore size of 62–490 µm and 56–437 µm for the Chi-Alg and Chi-Alg-fucoidan scaffolds, respectively. Negatively charged anionic fucoidan polymer interacted with chitosan and alginate. As a result, the pore size seemed to be decreased. Chi-Alg scaffolds showed well defined and interconnected pore structure, whereas the addition of fucoidan to the Chi-Alg composite resulted in reduced pore structure. The optimum pore size for bone tissue engineering remains unclear; however, investigations that sought to identify the optimum pore size for bone tissue engineering found pore sizes ranging from 80 to 500 μm to be viable [41]. The depicted pore size enables the scaffolds to allow for cell adhesion, proliferation and also nutrient supply, which will enable proper bone tissue growth. The optical microscopic images inferred that the dispersion of the components is uniform within the scaffolding network for both the Chi-Alg and Chi-Alg-fucoidan scaffolds.

Pukyong National University (PKNU).

### 2.8. Biocompatibility of the Scaffolds

**Figure 5:**
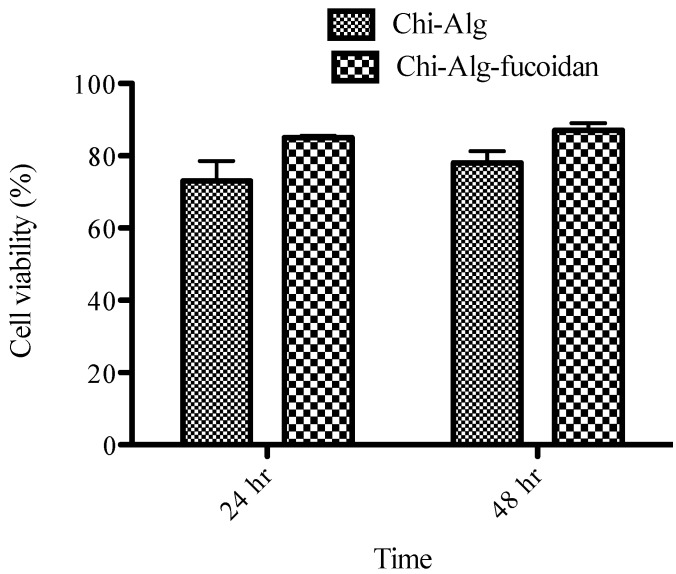
Cell viability of the Chi-Alg and Chi-Alg-fucoidan composite scaffolds with the MG-63 cell line.

The toxicity and biocompatibility of the prepared scaffolds are important concerns before proceeding to the *in vivo* study. An ample number of assays are available to measure the cytotoxicity of the scaffolds, such as the MTT (3-(4,5-dimethylthiazole-2-yl)-2,5-diphenyl tetrazolium), WSTs (Water Soluble Tetrazolium salts) and LDH (Lactate dehydrogenase) assays. In the present study, we have used the MTT assay to measure the biocompatibility of the prepared scaffolds. This assay is based on the ability of cellular mitochondrial dehydrogenase to reduce the yellow-colored tetrazolium salt to blue-colored formazan crystals. Human osteoblast-like cells (MG-63) were used in the experiment to measure the toxicity level of the prepared scaffolds. Cell viability on the fabricated scaffolds (Chi-Alg and Chi-Alg-fucoidan) at different time intervals is shown in Figure 5. The prepared scaffolds are shown to be biocompatible and non-cytotoxic in nature. There is no difference in the viability and cell proliferation between Chi-Alg and Chi-Alg-fucoidan scaffolds after 48 h, confirming that the addition of fucoidan in the Chi-Alg composite shows no cytotoxicity.

### 2.9. Alkaline Phosphatase Activity

**Figure 6:**
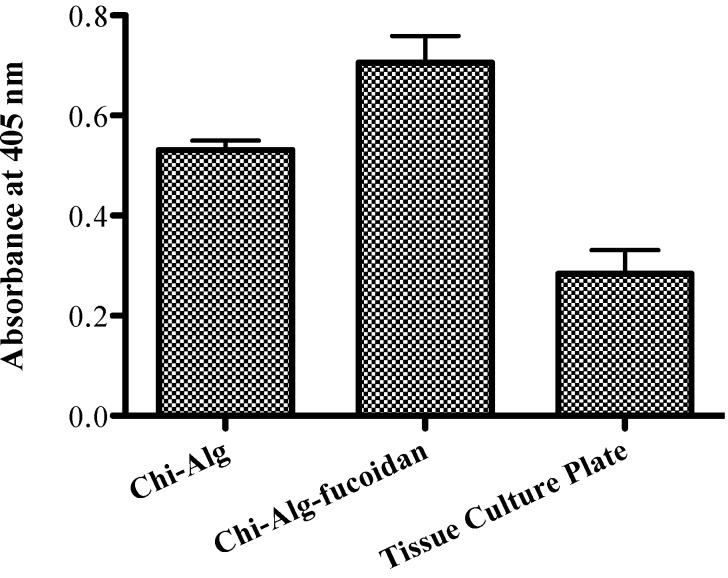
Alkaline phosphatase activity of the Chi-Alg and Chi-Alg-fucoidan scaffolds.

The measured ALP activity of the scaffolds are shown in Figure 6. Significantly, very little difference was observed in the ALP level among the composite scaffolds (the Chi-Alg and Chi-Alg-fucoidan scaffolds). It is known that fucoidan can significantly enhance the expression of osteogenesis-specific marker genes alkaline phosphatase and osteocalcin [31,32]. Korean researcher, Cho *et al.* (2009) reported that *Undaria pinnatifida*-derived fucoidan significantly induced the osteoblastic differentiation required for bone formation, by increasing the activity of the phenotypic markers, alkaline phosphatase and osteocalcin [30]. The understanding of the actual role of fucoidan in ALP is apprehensive; this might be due to the presence of a sulfated group in fucoidan.

### 2.10. Mineralization Results

**Figure 7:**
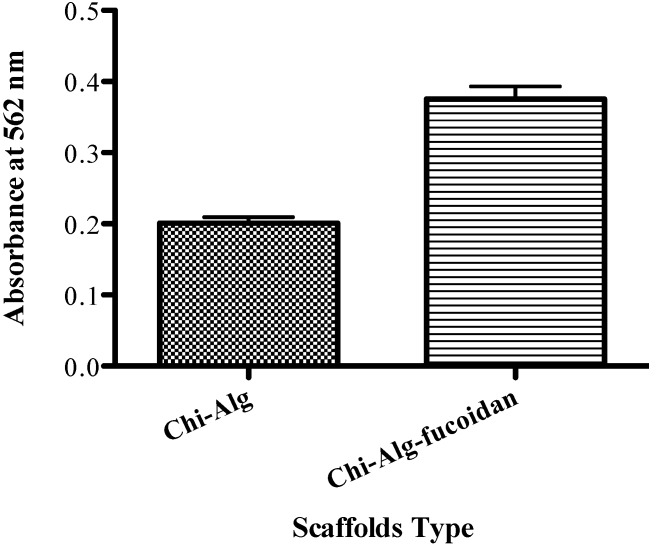
Quantitative measurement of minerals by the cetylpyridinium chloride (CPC) method: (**a**) Chi-Alg and (**b**) Chi-Alg-fucoidan.

The low molecular weight of fucoidan suggested that it has the capacity to promote osteoblast proliferation, increase fibrillar collagen content and induce mineralization, which is essential for bone tissue growth [31]. One hundred micrograms per milliliter of fucoidan increased the amount of HA in cells, which was detected by alizarin red S staining. The mineralization increased dose-dependently with fucoidan [30]. The mineralization effect of the Chi-Alg and Chi-Alg-fucoidan scaffolds on MG-63 cells is shown in Figure 7. Mineralization was increased by the presence of fucoidan in the Alg-Chi-fucoidan scaffolds as compared to the Chi-Alg scaffolds. According to our previous study, an increased ALP level and HA deposition were observed by the addition of fucoidan (100 μg/mL) in the osteoblast-like cells, which is an important process for bone tissue regeneration *in vivo* [30].

## 3. Experimental Section

### 3.1. Preparation of the Chitosan-Alginate (Chi-Alg) Scaffold

Alginate (3% w/v) was dissolved in 100 mL of water by using a mechanical stirrer (RW 20.n Lobortechik, Wasserburg, Germany) for 1 h to make a homogeneous solution. Secondly, chitosan (1% w/v, 310 kDa and 90%) was dissolved in 50 mL of 2% acetic acid solution and was carefully added into the alginate solution, with the help of a dropper. The homogeneous gel solution was stirred at 500 rpm for 1–2 h at room temperature. This gel solution was transferred into the tissue culture dish (35 × 10 mm^2^) and frozen at −24 °C for 1 day and freeze dried to form scaffolds. These scaffolds were immersed or cross-linked with 10% CaCl_2_ solution for 30 min, followed by soaking in absolute ethyl alcohol for 10 min. Finally, scaffolds were washed with an excess amount of water and freeze dried again for experimentation. In the present study, we have used Chi and Alg in a 3:1 weight ratio for the Chi-Alg scaffold fabrication.

### 3.2. Chitosan-Alginate-Fucoidan (Chi-Alg-Fucoidan) Scaffold

One hundred milligrams of fucoidan were added into the alginate solution, as prepared above, with the help of a dropper. This solution was mechanically stirred for 1 h to dissolve the entire fucoidan in the alginate solution to form a homogenous solution. Finally, chitosan (1% w/v) was dissolved in 50 mL of 2% acetic acid solution and was carefully added into the alginate-fucoidan solution, with the help of a dropper. This chitosan-alginate-fucoidan gel solution was transferred into the tissue culture dish, and the rest of the procedure was followed as described for the chitosan-alginate scaffold preparation. Ratios of Chi, Alg and Fucoidan of 3:1:0.1 weight were used in the construction of the Chi-Alg-fucoidan system.

### 3.3. Physicochemical Characterization

#### 3.3.1. Fourier Transform-Infrared (FT-IR) Spectroscopy

FT-IR was used to characterize and to know the chemical interactions between the Chi-Alg and Chi-Alg-fucoidan scaffolds. The spectra of the chitosan, alginate, fucoidan, Chi-Alg and Chi-Alg-fucoidan composite scaffolds were recorded using the KBr pellet method in an FT-IR spectrophotometer (Perkin Elmer, Waltham, MA, USA) with the range of 4000 cm^−1^ to 400 cm^−1^.

#### 3.3.2. Porosity Measurement

The total porosity was determined by the liquid displacement method. Initially, the volume of the ethanol and the dry weight of the scaffolds were measured. Then, the scaffold was immersed into the dehydrated alcohol for 48 h until absorbing the alcohol saturated it, and the scaffold was weighed again. Finally, the porosity of the sample was calculated based on the following formula:

Porosity = (V_2_ − V_1_ − V_3_)/(V_2_ − V_3_) × 100 (1)

where V_1_ = the initial known weight of the scaffold, V_2_ = the sum of the weights of ethanol and the submerged scaffold and V_3_ = the weight of ethanol after the removal of the scaffold.

Three parallel sets were analyzed for every scaffold, and the mean value of the porosities of different scaffolds was achieved.

#### 3.3.3. Water Uptake and Retention Abilities

The water uptake and retention ability of scaffold were studied, as described in our previous study [8,42]. Dry scaffolds were weighed (W_dry_) and immersed in 1× PBS solution for 24 h. Then, the scaffolds were gently removed from the beaker after 24 h and placed on a wire mesh rack. Excessive water was drained, and the scaffolds were weighed (W_wet_) after 5 min to determine the water uptake. To measure the water retention ability, the wet scaffolds were transferred to centrifuge tubes containing filter paper at the bottom, centrifuged (Combi 514-Hanil Science) at 500 rpm for 3 min and weighed immediately (W'_wet_). The percentages of water absorption (EA) and water retention (ER) of the scaffolds at equilibrium were calculated using the following equations:

EA = [(W_wet_ − W_dry_)/W_dry_] × 100 (2)

ER = [(W'_wet_ − W_dry_)/W_dry_] × 100 (3)

#### 3.3.4. Protein Adsorption Study

The protein adsorption ability of the Chi-Alg and Chi-Alg-fucoidan scaffolds was measured, as per a previous report [3]. Briefly, the scaffolds were equally cut into small pieces and placed in 24-well plates. The scaffolds were immersed in 100% ethanol for 1 h, and the ethanol was changed to 1× PBS for 30 min. After 30 min, the PBS was removed, and 500 µL of DMEM (Containing 10% FBS solution) were added to 24-well plates for 3 h, 12 h and 24 h to evaluate the protein adsorption amount corresponding to the different time periods. After a predetermined time, the scaffolds were blot dried and washed with 1× PBS 3 times to remove loosely adsorbed proteins. Proteins were agitated by incubating them in radioimmunoprecipitation (RIPA) buffer for 2 h. The eluted protein was measured by the absorbance at 570 nm using the bicinchoninic acid (BCA) protein assay method.

#### 3.3.5. *In Vitro* Biodegradation Behavior

The rate of degradation of the biocomposite scaffolds was studied according to the previous method [3]. The Chi-Alg and Chi-Alg-fucoidan scaffolds were equally weighed, and the initial weight was recorded as W_I_, followed by immersion in 1× PBS containing lysozyme (1000 U/L) and incubated at 37 °C at different intervals (24 and 72 h). After completion of the incubation period, the scaffolds were washed with deionized water to remove ions and blot dried with filter paper. The dry weights of the scaffolds were noted as (W_t_). The degradation was calculated by using the following formula:

Degradation = (W_I_ − W_t_)/W_I_ × 100 (4)

#### 3.3.6. Scanning Electron Microscopy (SEM) and Optical Microscopy

The pore size and surface morphology of the biocomposite scaffolds were studied using scanning electron microscopy (SEM JSM-6490LV, JEOL, Tokyo, Japan). Briefly, scaffold samples were cut into small pieces and fixed on carbon tape, then dried under vacuum and gold coated before examining under SEM. In addition, optical microscopy was also performed with the prepared scaffolds using an optical microscope (CTR 6000; Leica, Wetzlar, Germany).

### 3.4. Cell Culture Studies

#### 3.4.1. Cytotoxic Studies

The scaffolds (Chi-Alg and Chi-Alg-fucoidan) were cut into small pieces and placed in 24-well plates and incubated with cell culture media (DMEM) for 4 h at 37 °C in an incubator with 5% CO_2_ and 95% air. The MTT assay was used to measure the cytotoxicity of the prepared scaffolds. Osteosarcoma MG-63 cells with the concentration of 1 × 10^5^ cells/100 μL were seeded dropwise on the small pieces of scaffold and incubated at 37 °C. The cell culture media was removed on the respective days and incubated with fresh medium containing 200 μL of MTT (3-(4,5-dimethylthiazole-2-yl)-2,5-diphenyl tetrazolium) for 4 h in darkness. After the incubation period, the MTT dye was removed, followed by the addition of 200 μL DMSO to solubilize the formazan crystals, and optical densities (OD) were determined at 570 nm using a spectrophotometer (GENios R microplate reader; Tecan Austria GmbH, Grodig, Austria).

#### 3.4.2. Alkaline Phosphatase Activity

For ALP activity, scaffolds were immersed in 500 µL of osteogenic differentiation medium (ODM) in 24-well culture plate for 3 h, and 5 × 10^4^ cells/mL of MG-63 cells were seeded on the plate and kept for 7 days in the incubator. The ODM was replaced every 2 days. After the incubation, cells were rinsed with PBS, and 100 µL of 25 mM carbonate buffer (pH 10.3) containing 0.2% Tritox X-100 were added. Each well of the plate was transferred with 50 µL of 250 mM carbonate buffer (pH 10.3) containing 2.5 mM MgCl_2_ and 15 mM *p*-nitro phenyl phosphate (*p*-NPP). The plate was incubated for 30 min at 37 °C, and the absorbance was measured at 405 nm in a spectrophotometer (Tecan Austria GmbH, Grodig, Austria).

#### 3.4.3. Mineralization Assay

The mineralization assay was performed by alizarin red S stain. Briefly, scaffolds were immersed in ODM in a 24-well plate for 3 h. After 3 h, MG-63 cells were seeded dropwise into the plate (5 × 10^3^ cells/100 µL). The media were changed every 2 days until 14 days, then the media were removed. The cells were washed twice by PBS and fixed in 70% ethyl alcohol for 1 h at room temperature. The ethyl alcohol-fixed cells and scaffolds were stained with 40 mM alizarin red S (pH 4.1) for 10 min. Cells were washed with deionized water five times and with PBS two times, then the cells were incubated in 10 mM of sodium phosphate buffer (pH 7.0) containing 10% of cetylpyridium chloride for 15 min. The optical density was measured at 562 nm using a GENios R microplate reader (Tecan Austria GmbH, Grodig, Austria).

### 3.5. Statistical Analysis

All the data are expressed as means ± the standard deviation of a minimum of three replicates for each scaffold in each experiment, using Graphpad Prism 5.0.

## 4. Conclusions

In this study, we prepared two different types of composite scaffolds. Chemical ionic interactions were observed between chitosan, alginate and fucoidan, which led to the improved bioactivity of the scaffolds. The addition of negatively charged and sulfated fucoidan in Chi-Alg showed a better activity towards bone tissue regeneration. Owing to the great water uptake ability, sufficient porosity, enhanced protein adsorption and increased mineralization effects, the Chi-Alg-fucoidan scaffolds would be promising biomaterials for bone tissue engineering.
